# Ultrasound‐guided posterior lateral branches steroid injections of the sacral foramina for chronic new‐onset sacroiliac joint pain management after spinal fusion surgery: A prospective study of single‐center in Vietnam

**DOI:** 10.1002/kjm2.12867

**Published:** 2024-05-31

**Authors:** Tuan Anh Pham, Anh Minh Nguyen, Phuc Long Nguyen, Viet‐Thang Le

**Affiliations:** ^1^ Faculty of Medicine University of Medicine and Pharmacy at Ho Chi Minh Ho Chi Minh City Vietnam; ^2^ Department of Neurosurgery Nguyen Tri Phuong Hospital Ho Chi Minh Vietnam; ^3^ Department of Neurosurgery University Medical Center at Ho Chi Minh City Ho Chi Minh City Vietnam

Lumbar spinal fusion (LSF) surgery is increasingly popular for treating degenerative spinal pain, but studies reveal failure rates of 5%–30%. Persistent or new low back pain post‐surgery is common, often due to adjacent segment disease, sacroiliac joint (SIJ) pain, or inappropriate instrumentation. SIJ pain alone affects 16.2%–43% of cases, significantly impacting their quality of life.^1^ Current post‐LSF SIJ pain management includes medication, physical therapy, invasive interventions, and surgery. Nerve blocks targeting sacral dorsal rami branches using ultrasound‐guided injection techniques are gaining attention, prized for accessibility and cost‐effectiveness. Our study evaluates the efficacy of this method.

We conducted a 2‐year prospective study involving 56 cases of SIJ pain after LSF. The selection criteria were in line with North American Spine Society guidelines, including unilateral SIJ pain with a Visual Analogue Scale (VAS) ≥5, localized tenderness at Fortin's point, positive Fortin sign, at least three positive SIJ pain tests (Patrick test, Gaenslen test, etc.), and unsuccessful conservative treatment for at least 6 months. Exclusion criteria comprised symptomatic SIJ conditions such as inflammatory, tumoral, traumatic, or infectious disease.^2^

During the procedure, each lateral branch block (LBB) included 1 mL of dexamethasone 4 mg and 1 mL of bupivacaine 0.5%, administered via a 5‐inch, 22‐G spinal needle. Under sonography, the lateral sacral crest was targeted using the landmarks of the posterior sacral foramen and the first three transverse tubercles. Three needles were placed per transverse tubercle from S1 to S3 using the in‐plane approach, positioned at the periosteum level within 1 cm lateral from the lateral border of the posterior sacral foramen.^3^

Post‐procedural, improvement was accessed using VAS, Oswestry Disability Index (ODI), and MacNab criteria, a reduction of 2 points or ≥50% in the VAS was considered a response to treatment.

Among the 61 patients, with a mean age of 53.5 and predominantly female, the average VAS decreased from 2.25 ± 0.82 at discharge to 1.11 ± 0.41 at 1 month and 1.24 ± 0.63 at 6 months post‐intervention. Similarly, the average ODI improved from 23.5 ± 6.1 at discharge to 21.3 ± 4.4 at 1 month and 22.1 ± 3.5 at 6 months (Table 1). The response rate, as per the ODI index, declined slightly from 88.5% to 86.9% over 6 months. At 6 months post‐SIJ injection, the more levels of fusion, the poorer the response rate was noted. Patients with S1 fusion had a worse response compared to those without sacral fusion, though this difference was not statistically significant. Regarding modified MacNab criteria, most patients experience excellent outcomes, supporting the efficacy of LBB. The common side effects were pain at the injection site (3.3%) and transient headaches (6.5%).

**TABLE 1:** Post‐procedural outcomes.

	Before‐procedural	Post‐procedural		
		Discharge	1 month	6 months
ODI scores	59.1 ± 13.6	23.5 ± 6.1	21.3 ± 4.4	22.1 ± 3.5
Response to treatment and level of fusion (cases)				
1 level (*N* = 1)	1	1 (100%)	1 (100%)	1 (100%)
2 levels (*N* = 32)	32	32 (100%)	28 (87.5%)	26 (81.3%)
3 levels (*N* = 18)	18	15 (83.3%)	12 (66.7%)	9 (50.0%)
4 levels (*N* = 10)	10	10 (100%)	6 (60%)	2 (20%)
Response to treatment and S1 fusion (cases)				
Include S1 (*N* = 31)	31	29 (93.5%)	28 (91.4%)	27 (87.1%)
Not include S1 (*N* = 30)	30	28 (93.3%)	26 (86.7%)	26 (86.7%)
Modified Macnab criteria at 6 months				
Excellent				24 (39.3%)
Good				31 (50.8%)
Fair				6 (9.9%)
Poor				0 (0%)

Our study emphasizes the burden of SIJ pain post‐LSF. Older age and female gender correlate with higher SIJ pain likelihood.^4^ SIJ injection provides significant pain relief, surpassing conservative treatments, and improving patient quality of life. The number of fusion levels accelerates SIJ degeneration and reduces treatment efficacy, and S1 sacral fusion shows poorer pain relief, consistent with the literature.^5^

## CONFLICT OF INTEREST STATEMENT

The authors declare no conflicts of interest.
